# The low-lethal concentrations of rotenone and pyrethrins suppress the population growth of *Rhopalosiphum padi*

**DOI:** 10.1038/s41598-024-67286-1

**Published:** 2024-07-17

**Authors:** Li Xu, Zhenghua Wu, Jingkun Li, Yihao Xu, Feng Zhou, Fulong Zhang, Dongzhi Li, Lin Zhou, Runqiang Liu

**Affiliations:** 1 Henan Engineering Research Center of Green Pesticide Creation & Intelligent Pesticide Residue Sensor Detection, Henan Institute of Science and Technology, Xinxiang, 453003 China; 2 College of Resources and Environment, Henan Institute of Science and Technology, Xinxiang, 453003 China; 3 Beijing Kingbo Biotechnology Co., Ltd, Beijing, 101399 China; 4 Plant Protection College, Henan Agricultural University, Zhengzhou, 450046 China

**Keywords:** *Rhopalosiphum padi*, Rotenone, Pyrethrins, Sublethal effect, Population growth, Agroecology, Community ecology

## Abstract

As an important pest on winter wheat, *Rhopalosiphum padi* (L.) causes damage to the wheat yield by sucking plant nutrients, transmitting plant viruses and producing mildew. *R. padi* has been reported to develop resistance to pyrethroids and neonicotinoids. To explore potential alternative approaches for *R. padi* control, the activity of 10 botanical insecticides was evaluated. Results suggested that the toxicity of rotenone and pyrethrins to *R. padi* were the highest and near to the commonly used chemical insecticides. When exposed to the low-lethal concentrations (LC_10_, LC_30_) of rotenone or pyrethrins for 24 h, the lifespan and fecundity of adults in F0 generation decreased significantly compared to control. The negative effect could also be observed in the F1 generation, including the decreased average offspring, longevity of adult, and prolonged nymph period. The population parameters in F1 generation of *R. padi* were also inhibited by exposing to the low-lethal concentrations of rotenone or pyrethrins, including the decreased net reproductive rate, intrinsic rate of natural increase, finite rate of population increase, and gross reproduction rate. Co-toxocity factor results showed that mixtures of rotenone and thiamethoxam, pyrethrins and thiamethoxam showed synergistic effect. Our work suggested that rotenone and pyrethrins showed negative effect on the population growth under low-lethal concentrations. They are suitable for *R. padi* control as foliar spraying without causing population resurgence.

## Introduction

Aphids are important sucking pests feeding on agricultural crops in tropical and subtropical regions. *Rhopalosiphum padi* (L.) is regarded as one of the predominant species infesting wheat^1^, the main staple crop worldwide. It causes severe damage to the yields of wheat by sucking plant nutrients directly, and transmitting plant viruses, including the barley yellow dwarf virus. The increasing of global warming conditions and the intensive agriculture, monocultures provided suitable conditions for aphid population growth^2^, thus leading to annually increased damage caused by aphids^3,4^.

In China, Huang-Huai-Hai plain is the major planting area of winter wheat, and the control of *R. padi* mainly relies on the insecticide foliar spraying in early May. A total of 40 cases reported *R. padi* resistance to pyrethroids, organophosphates and neonicotinoids (https://www.pesticideresistance.org/display.php?page=species&arId=369). Botanical insecticides are compounds derived from the plants. They have been shown less damage to environment and have been not easier to develop resistance than chemical insecticides. Screening botanical insecticides with high toxicity to *R. padi* and alternatively application could manage resistance to chemical insecticides and decrease the application dosage of chemical insecticides. There are multiple botanical pesticides in China, and some of them show both insecticide and fungicide activities. It is necessary to evaluate the toxicity of the botanical insecticides to *R. padi* and thus screen out some valuable cases*.*

Rotenone, pyrethrins, matrine, azadirachtin are well reported botanical insecticides showing broad and high toxicity to insect pests and registered for insect control in China (http://www.chinapesticide.org.cn/zwb/dataCenter). Multiple botanical chemicals have been reported to show potential in insect control. Torres et al^5^ reported that resveratrol showed growth regulatory activity against *Spodoptera frugiperda* (J.E. Smith). Osthole and its derived chemicals showed insecticide activity to *Mythimna separata* (Walker), *Plutella xylostella* (Linnaeus), *Myzus persicae* (Sulzer)^6,7^. Carvacrol showed larvicidal and ovicidal activity to *Helicoverpa armigera* (Hübner), *Alphitobius diaperinus* (Panzer)^8,9^, *Lycoriella ingenua* (Dufour)^10^, *Odontotermes assamensis* (Holmgren)^11^, *Blattella germanica* (Linnaeus)^12^, *Spodoptera litura* (Fabricius)^13^, *Reticulitermes speratus* (Kolbe)^14^, *Aedes aegypti* (Linnaeus)^15^. Eugenol showed persistent larvicidal activity to *A. aegypti* after selection for 30 generations continuously^16^. Besides, eugenol was reported to be effective in fire ant repellent^17^. Eucalyptol showed strong contact toxicity against the larvae of *P. xylostella* and fumigant activity against the adults of *P. xylostella*^18^, *Tribolium castaneum* (Herbst), and antifeedant activity to *T. castaneum*^19^.

Traditionally, acute toxicity was used to evaluate the effects of insecticides to pests. Besides the direct mortality caused by acute toxicity, individuals survived from exposure to the sublethal concentrations of insecticides also sustain impairments on the growth or fecundity in the parental generation or the offsprings, which might show influence on the life table or population growth^20–22^. As the botanical insecticide deposits quickly degrade over time leading to prevailing low-lethal exposure, the sublethal effect of botanical insecticide may be as important or even more important than mortality^23^. The sublethal effects of insecticides on pests need to be evaluated because it will provide practical information for integrated pest management.

In this study, the botanical pesticides with high toxicity to *R. padi* were screened out. To make reasonable application strategies of botanical insecticide, the sublethal effect of these insecticides on the biological traits and population growth of *R. padi* were evaluated. The synergist mixtures containing botanical insecticide and chemical insecticide were screened to increase the control effect and decrease the application dosage of chemical insecticide. The results laid foundation for the integrated control of *R. padi*.

## Results

### Rotenone and pyrethrins showed high toxicity to *R. Padi*

A total of 10 botanical pesticides and 2 commonly used chemical insecticides were selected to determine their toxicity to the adults of *R. Padi.* Among the botanical pesticides, the LC_50_ values of rotenone and pyrethrins to *R. Padi* were the lowest, which were 10.091 and 5.161 mg L^−1^, respectively after treated for 24 h, while 7.146 and 3.403 mg L^−1^, respectively after treated for 48 h (Table 1). While the LC_50_ values of the other botanical pesticides were between 171.597 and 5993.123 mg L^−1^, indicating their low contact toxicity. The LC_50_ values of the chemical insecticides, bifenthrin and thiamethoxam, were 3.028 and 16.447 mg L^−1^ after treated for 24 h, respectively (Table 1). The contact toxicity of rotenone and pyrethrins was near to that of commonly used chemical insecticides.

**Table 1:** Toxicity of botanical insecticides and chemical insecticides to *R. padi.*

Insecticides	TAT^a^	Regression equation	LC_50_ (mg L^−1^, 95%FL)	Correlation coefficient	Chi-square (χ2)
Pyrethrins	24 h	y = − 0.830 + 1.165x	5.161(3.316–9.380)	0.955	2.425
	48 h	y = − 0.876 + 1.647x	3.403(2.101–5.089)	0.913	4.811
Rotenone	24 h	y = − 1.513 + 1.507x	10.091(7.255–13.695)	0.968	1.606
	48 h	y = − 1.418 + 1.660x	7.146(5.068–9.566)	0.985	3.185
Azadirachtin	24 h	y = − 2.229 + 0.966x	203.549(119.174–720.621)	0.885	2.191
	48 h	y = − 2.200 + 0.959x	197.119(110.684–828.595)	0.900	2.033
Matrine	24 h	y = − 4.304 + 1.466x	862.999(581.970–1349.854)	0.995	0.191
	48 h	y = − 2.479 + 1.109x	171.597(93.438–275.835)	0.988	0.443
Carvacrol	24 h	y = − 26.681 + 7.648x	3078.603(2392.132–3997.955)	0.870	8.711
	48 h	–^b^	–	–	–
Eugenol	24 h	y = − 25.301 + 7.145x	3476.446(2350.364–4246.429)	0.937	7.219
	48 h	y = − 16.124 + 4.573x	3359.489(1931.959–4443.940)	0.980	10.879
Citronellal	24 h	y = − 35.007 + 9.786x	3777.999(1649.826–4815.431)	0.877	14.048
	48 h	–	–	–	–
Osthole	24 h	y = − 32.583 + 8.904x	4563.730(4260.472–4840.934)	0.956	4.140
	48 h	y = − 43.261 + 11.857x	4452.480(4099.247–4722.612)	0.996	0.870
Resveratrol	24 h	y = − 24.298 + 6.541x	5188.651(435.072–6893.853)	0.927	5.549
	48 h	y = − 27.791 + 7.541x	4844.630(4383.542–5266.572)	0.916	5.259
Eucalyptol	24 h	y = − 65.060 + 17.222x	5993.123(5744.231–6229.424)	0.950	1.386
	48 h	y = − 73.005 + 19.367x	5883.373(5595.710–6118.891)	0.992	0.637
Bifenthrin	24 h	y = − 0.785 + 1.632x	3.028(2.305–4.193)	0.923	5.349
Thiamethoxam	24 h	y = − 1.420 + 1.168x	16.447(7.453–26.563)	0.936	3.071

^a^Time after treatment.

^b^Indicated data not available.

The low-lethal concentrations of rotenone and pyrethrins to *R. Padi* were also obtained after treated for 24 h. The LC_10_ and LC_30_ values of rotenone were 1.424 and 4.529 mg L^−1^, respectively, and 0.410 and 1.830 mg L^−1^, respectively for pyrethrins (Table 2).

**Table 2:** The LC_10_ and LC_30_ values of rotenone and pyrethrins to *R. padi*.

Insecticides	TAT^a^	LC_10_ (mg L^−1^, 95% FL)	LC_30_ (mg L^−1^, 95% FL)
Rotenone	24 h	1.424 (0.622–2.390)	4.529 (2.786–6.390)
pyrethrin	24 h	0.410 (0.125–0.778)	1.830 (1.032–2.844)

^a^Time after treatment.

### Exposure to the low-lethal concentrations of rotenone decreased the population growth of *R. padi* and increased the development duration of nymph

After exposed to rotenone at LC_10_ or LC_30_ for 24 h, the adults of *R. padi* were kept on fresh wheat leaves until death. Compared with adults exposed to 0.1% Tween 80, the average lifespan and fecundity decreased significantly in adults exposed to LC_30_ rotenone, and the decrease rate was 17.74% and 19.54%, respectively (Table 3). There was no significant difference of average lifespan and fecundity between adults exposed to 0.1% Tween 80 and LC_10_ rotenone, or LC_10_ and LC_30_ rotenone (Table 3).

**Table 3:** The sublethal effect of rotenone on the lifespan and fecundity of F0 generation of *R. padi.*

Low-lethal concentration	The average lifespan (day)	Average offspring
CK	10.88 ± 2.17 a	18.83 ± 4.27 a
LC_10_	9.48 ± 2.17 ab	17.74 ± 3.85 ab
LC_30_	8.95 ± 2.33 b	15.15 ± 4.49 b

Different lowercase letters in a column indicated significant differences analyzed by the one-way ANOVA with Turkey’s HSD test (*P* < 0.05).

The low-lethal concentrations of rotenone showed significant sublethal negative effect on the development duration and fecundity in the F1 generation of *R. padi*. Although the F1 generation of *R. padi* were kept on fresh wheat leaves since they were new-born, *R. padi* derived from parental generation exposed to LC_30_ rotenone showed significantly longer development duration of nymphs and fewer offspring when compared with that derived from parental generation exposed to 0.1% Tween 80 (Table 4). After exposed to rotenone at LC_10_ or LC_30_, the longevity of adults in the F1 generation showed no change. Exposure to LC_10_ rotenone also prolonged the development duration of nymph significantly than that in control, while the average offspring showed no significant difference with that in control (Table 4).

**Table 4:** The sublethal effect of rotenone on the longevity and mean fecundity of adults in F1 generation of *R. padi.*

Low-lethal concentration	Development duration of nymph (day)	Adult longevity (day)	Average produced offspring
CK	4.43 ± 0.51 a	9.09 ± 1.56 a	19.43 ± 5.13 a
LC_10_	4.92 ± 0.76 b	8.00 ± 1.91 a	17.04 ± 6.11 a
LC_30_	4.95 ± 0.72 b	8.05 ± 1.70 a	13.27 ± 3.91 b

Different lowercase letters in a column indicated significant differences analyzed by the one-way ANOVA with Turkey’s HSD (*P* < 0.05).

The population parameters of *R. padi* in the F1 generation were calculated. The values of r_m_ and λ in LC_10_ and LC_30_ groups decreased significantly in the F1 generation than that of control, while they showed no significant difference between LC_10_ and LC_30_ groups. The values of R_0_ and GRR in LC_30_ group decreased significantly than that of control or LC_10_ group, while they showed no significant difference between LC_10_ and control groups. The values of T in LC_10_ or LC_30_ group showed no change with that of control (Table 5).

**Table 5:** The sublethal effect of rotenone on the population parameters in F1 generation of *R. padi.*

Low-lethal concentration	T (day)	R_0_	r_m_ (day^-1^)	λ (day^-1^)	GRR
CK	7.8082 ± 0.1226 a	19.3913 ± 1.0541 a	0.3797 ± 0.0069 a	1.4619 ± 0.0101 a	20.0602 ± 1.0816 a
LC_10_	8.1335 ± 0.2172 a	17.2000 ± 1.1690 a	0.3498 ± 0.0104 b	1.4188 ± 0.0148 b	19.1425 ± 1.2625 a
LC_30_	7.7715 ± 0.2111 a	13.2727 ± 0.8130 b	0.3327 ± 0.0094 b	1.3948 ± 0.0130 b	14.1305 ± 0.7522 b

Different lowercase letters in a column indicated significant differences analyzed by the one-way ANOVA with Turkey’s HSD test (*P* < 0.05).

**Figure 1:**
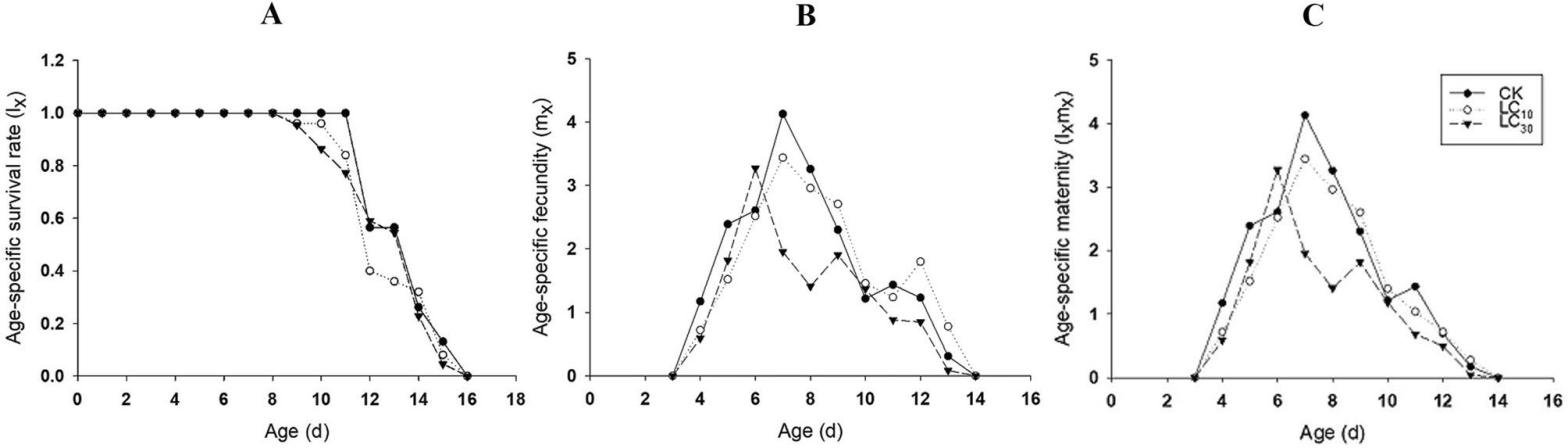
Age-specific survival rate (l_x_), fecundity (m_x_), net maternity (l_x_m_x_) of *R. padi* exposed to control (**A**), LC_10_ (**B**) and LC_30_ (**C**) of rotenone.

After exposed to the low-lethal concentrations of rotenone, the population dynamics of *R. padi* were estimated. The l_x_ in the F1 individuals showed CK > LC_10_ > LC_30_ during 8–11 d and 15–16 d (Fig. 1A). The highest peak of m_x_ in the control group was 4.13 offspring/female occurring at age of 7 d. While the highest peak of m_x_ in LC_10_ group was 3.44 offspring/female occurring at age of 7 d. The highest peak of m_x_ in LC_30_ group appeared at age of 6 d with 3.1 offspring/female, ahead of control (Fig. 1B). The l_x_m_x_ curves showed a similar trend with that of m_x_ (Fig. 1C).

**Figure 2:**
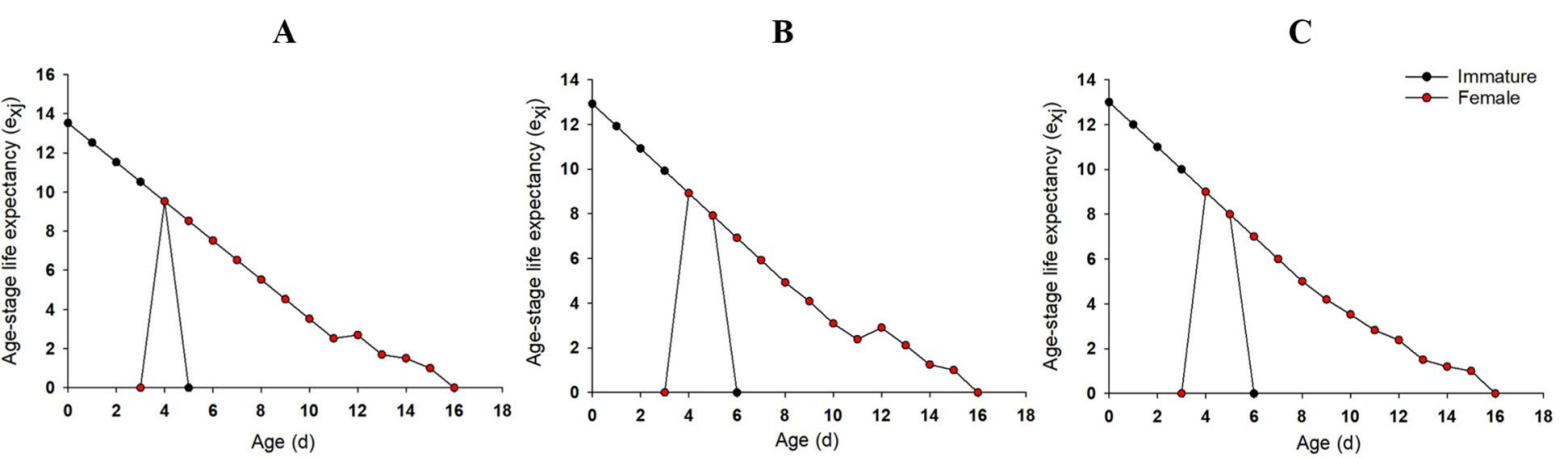
Age-stage-specific life expectancy (e_xj_) of *R. padi* exposed to control (**A**), LC_10_ (**B**) and LC_30_ (**C**) of rotenone.

The e_xj_ indicate the expected life-span of individuals at age x and stage j after age x. The e_xj_ of immature and adult stages for the aphids treated with rotenone at LC_10_ and LC_30_ were slightly lower than those of control (Fig. 2). For example, the e_xj_ of the new born nymph in LC_10_ and LC_30_ rotenone group was 12.92 d and 13.00 d, respectively, lower than control (13.52 d, Fig. 2). While e_xj_ of newly molted adult in LC_10_ and LC_30_ rotenone was 8.92 d and 9.00 d, respectively, lower than control (9.52 d, Fig. 2).

**Figure 3:**
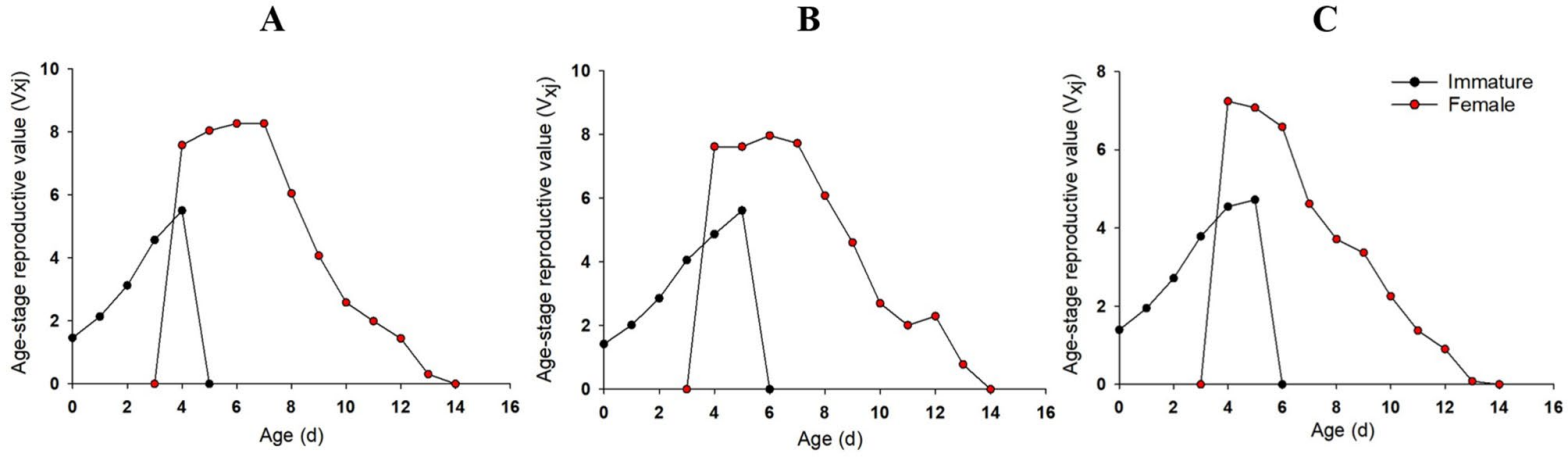
Age-stage-specific reproductive value (V_xj_) of *R. padi* exposed to control (**A**) LC_10_ (**B**) and LC_30_ (**C**) of rotenone.

The V_xj_ curve of female aphids showed a trend of first increasing and then decreasing (Fig. 3). However, the reproductive peak of the offspring in the LC_10_ and LC_30_ treatment groups reached 7.96 offsprings on the 6th day and 7.23 offsprings on the 4th day post-treatment, respectively, which were lower than those in the control group, reaching a reproductive peak of 8.26 offsprings on the 7th day post-treatment (Fig. 3).

### Exposure to the low-lethal concentrations of pyrethrins decreased the population growth of *R. padi* and increased the development duration of nymph

Exposed to pyrethrins at LC_30_ for 24 h significantly decreased the average lifespan and fecundity of adults when compared to the control group. Exposed to pyrethrins at LC_10_ for 24 h significantly decreased the average lifespan and showed no influence on the fecundity of adults when compared with that of control. There was no significant difference of lifespan and fecundity between adults exposed to LC_10_ and LC_30_ pyrethrins (Table 6).

**Table 6:** The sublethal effect of pyrethrins on the fecundity and lifespan of F0 generation of *R. padi.*

Low-lethal concentration	The average lifespan (day)	Average offspring
CK	10.88 ± 2.17 a	18.83 ± 4.27 a
LC_10_	8.68 ± 1.41 b	15.96 ± 5.11 ab
LC_30_	8.40 ± 1.66 b	14.56 ± 4.06 b

Different lowercase letters in a column indicated significant differences analyzed by the one-way ANOVA with Turkey’s HSD (*P* < 0.05).

Compared with control, exposed to LC_30_ of pyrethrins showed significant increase in the development duration of nymph, and inhibition in the longevity of adults and the fecundity of *R. padi* in the F1 generation. Exposed to LC_10_ of pyrethrins significantly decreased the fecundity compared to the control, while showed no influence on the development duration of nymph or adults (Table 7). There was no significant difference between these parameters of *R. padi* derived from LC_10_ and LC_30_ groups.

**Table 7:** The sublethal effect of pyrethrins on the longevity and mean fecundity of adults in F1 generation of *R. padi.*

Low-lethal concentration	Development duration of nymph (day)	Adult longevity (day)	Average produced offspring
CK	4.43 ± 0.51 a	9.09 ± 1.56 a	19.43 ± 5.13 a
LC_10_	4.86 ± 0.64 ab	8.05 ± 1.62 ab	15.36 ± 5.88 b
LC_30_	4.63 ± 0.65 b	7.88 ± 1.26 b	12.04 ± 3.65 b

Different lowercase letters in a column indicated significant differences analyzed by the one-way ANOVA with Turkey’s HSD (*P* < 0.05).

The values of R_0_, r_m_, λ and GRR in LC_10_ and LC_30_ groups decreased significantly in the F1 generation than that of control. The values of T increased significantly in LC_10_ than those in LC_30_ and control groups (Table 8). The values of R_0_ and GRR in LC_30_ group decreased significantly than that in LC_10_ group, while r_m_, λ, T showed no significant change than that in LC_10_ group.

**Table 8:** The sublethal effect of pyrethrins on the population parameters in F1 generation of *R. padi.*

Low-lethal concentration	T (day)	R_0_	r_m_ (day^−1^)	λ (day^-1^)	GRR
CK	7.7725 ± 0.1267 b	19.3913 ± 1.0443 a	0.3815 ± 0.0071 a	1.4644 ± 0.0104 a	20.0602 ± 1.0700 a
LC_10_	8.4307 ± 0.2271 a	15.3636 ± 1.2247 b	0.3241 ± 0.0118 b	1.3827 ± 0.0163 b	16.2540 ± 1.1397 b
LC_30_	7.6299 ± 0.2047 b	12.0417 ± 0.7315 c	0.3261 ± 0.0108 b	1.3856 ± 0.0150 b	12.8076 ± 0.9013 c

Different lowercase letters in a column indicated significant differences analyzed by the one-way ANOVA with Turkey’s HSD test (*P* < 0.05).

**Figure 4:**
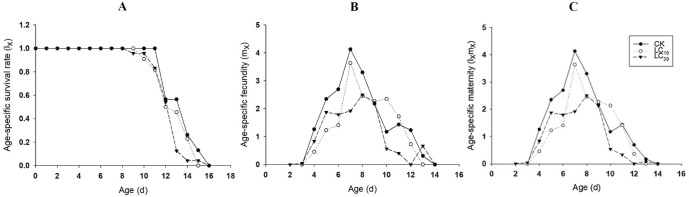
Age-specific survival rate (l_x_), fecundity (m_x_), net maternity (l_x_m_x_) of *R. padi* exposed to control (**A**), LC_10_ (**B**) and LC_30_ (**C**) of pyrethrins.

The l_x_ in the F1 individuals of control groups was obviously higher than that in LC_10_ and LC_30_ pyrethrins treatment groups during 8–12 d. After 12 d, the l_x_ showed a trend of CK > LC_10_ > LC_30_ (Fig. 4A). Compared with the highest peak of m_x_ in the control group (4.13 offspring/female at the 7th d), that in LC_10_ and LC_30_ groups decreased to 3.64 offspring/female at the 7th d and 2.10 offspring/female at the 8th d (Fig. 4B). The l_x_m_x_ curves showed a similar trend to that of m_x_ (Fig. 4C).

**Figure 5:**
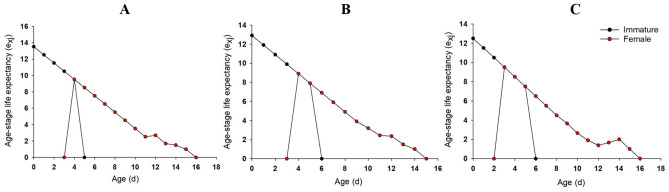
Age-stage-specific life expectancy (e_xj_) of *R. padi* exposed to control (**A**), LC_10_ (**B**) and LC_30_ (**C**) of pyrethrins.

The e_xj_ of immature and adult stages for the aphids treated by pyrethrins at LC_10_ and LC_30_ were slightly lower than those of the control (Fig. 5). For example, the e_xj_ of the new born nymph in the LC_10_ and LC_30_ pyrethrins group were 12.91 d and 12.50 d, respectively, lower than that in the control (13.50 d, Fig. 5). While e_xj_ of newly molted adult in the LC_10_ and LC_30_ pyrethrins group were 8.91 d and 8.50 d, respectively, lower than that in the control (9.52 d, Fig. 5). The immature stage in LC_10_ and LC_30_ group were 6 d, delayed for 1 d than that of control. The adult stage in LC_30_ group began from the 2nd d, earlier than the 3^rd^ d in LC_10_ and control groups.

**Figure 6:**
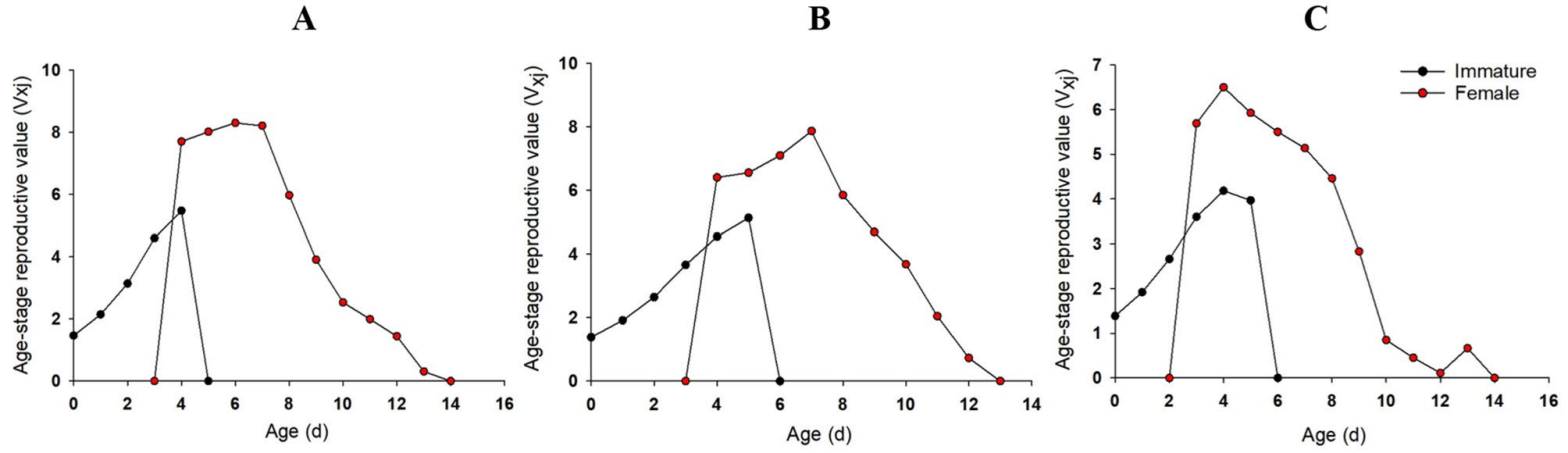
Age-stage-specific reproductive value (V_xj_) of *R. padi* exposed to control (**A**), LC_10_ (**B**) and LC_30_ (**C**) of pyrethrins.

The reproductive peak of the V_xj_ curve of immature aphids in control, LC_10_ and LC_30_ groups appeared at the 4th, 5th and 4th d, respectively, while 6th, 7th, 4th for that in female aphids. The reproductive peak of the V_xj_ curve of immature aphids in control, LC_10_ and LC_30_ groups were 5.47, 5.13 and 4.18 offsprings, respectively, while 8.30, 7.87, 6.49 offsprings for female aphids (Fig. 6).

### Screening of the synergist mixture containing rotenone or pyrethrins and chemical insecticides to *R. padi*

The co-toxicity factor method was used to screen the synergist mixtures containing rotenone or pyrethrins and thiamethoxam or bifenthrin. Among the set ratios, synergist mixtures were obtained in all the combinations except for the mixtures of pyrethrin and bifenthrin at 1/2 LC_50_. The combination of rotenone and thiamethoxam showed the best synergist effect, as four out of five ratios (1:5, 1:2, 1:1, 5:1) prepared with LC_50_ or 1/2 LC_50_ showed synergist (Table 9). The combination of rotenone and bifenthrin prepared with LC_50_ and 1/2 LC_50_ both showed synergist at the ratio of 1:5. The mixture of pyrethrins and thiamethoxam prepared with LC_50_ showed synergist for all the ratios, while synergist was screened out only for ratios 1:5, 1:2, 1:1 prepared with 1/2 LC_50_. The mixture of pyrethrins and bifenthrin prepared with LC_50_ showed synergist for ratios 1:5, 1:2, 1:1 (Table 9).

**Table 9:** The synergist mixtures screening using the co-toxicity factor method.

	Mixture	Ratio	Mortality obtained from LC-P regression (%)		Expected mortality (%)	Observed mortality (%)	Co-toxicity factor	Results
			A	B				
LC_50_ A + B	Rotenone + Bifenthrin	1:5	12.06	43.05	55.11	73.17	32.77	Synergist
		1:2	23.64	38.74	62.38	85.37	36.85	Synergist
		1:1	32.51	31.16	63.67	78.05	22.58	Synergist
		2:1	39.56	21.84	61.40	65.85	7.25	Additive
		5:1	45.33	10.25	55.58	65.85	18.48	Additive
	Rotenone + Thiamethoxam	1:5	12.06	46.34	58.40	95.12	62.88	Synergist
		1:2	23.64	41.85	65.49	92.68	41.52	Synergist
		1:1	32.51	36.32	68.83	82.93	20.48	Synergist
		2:1	39.56	28.95	68.51	75.61	10.36	Additive
		5:1	45.33	18.25	63.58	78.05	22.76	Synergist
	Pyrethrins + Bifenthrin	1:5	18.25	43.05	61.30	74.42	21.40	Synergist
		1:2	28.92	38.74	67.66	83.72	23.74	Synergist
		1:1	36.35	31.16	67.51	86.05	27.46	Synergist
		2:1	41.92	21.84	63.76	53.49	− 16.11	Additive
		5:1	46.34	10.25	56.59	67.44	19.18	Additive
	Pyrethrins + Thiamethoxam	1:5	18.25	46.34	64.59	90.70	40.42	Synergist
		1:2	28.92	41.85	70.77	90.70	28.16	Synergist
		1:1	36.35	36.32	72.67	97.67	34.41	Synergist
		2:1	41.92	28.95	70.87	93.02	31.26	Synergist
		5:1	46.34	18.25	64.59	90.70	40.42	Synergist
1/2 LC_50_ A + B	Rotenone + Bifenthrin	1:5	5.21	26.74	31.95	44.19	38.31	Synergist
		1:2	12.03	21.71	33.74	20.93	− 37.97	Antagonism
		1:1	18.22	16.23	34.45	16.28	− 52.74	Antagonism
		2:1	23.64	10.21	33.85	23.26	− 31.29	Antagonism
		5:1	28.42	4.04	32.46	9.30	− 71.35	Antagonism
	Rotenone + Thiamethoxam	1:5	5.21	32.85	38.06	86.05	126.09	Synergist
		1:2	12.03	28.96	40.99	90.70	121.27	Synergist
		1:1	18.22	24.21	42.43	79.07	86.35	Synergist
		2:1	23.64	18.23	41.87	83.72	99.95	Synergist
		5:1	28.42	10.43	38.85	53.49	37.68	Synergist
	Pyrethrins + Bifenthrin	1:5	10.43	26.74	37.17	39.02	4.98	Additive
		1:2	18.23	21.71	39.94	39.02	− 2.30	Additive
		1:1	24.14	16.23	40.37	2.44	− 93.96	Antagonism
		2:1	28.96	10.21	39.17	29.27	− 25.27	Antagonism
		5:1	32.94	4.04	36.98	− 2.44	− 106.60	Antagonism
	Pyrethrins + Thiamethoxam	1:5	10.43	32.85	43.28	87.80	102.87	Synergist
		1:2	18.23	28.96	47.19	75.61	60.22	Synergist
		1:1	24.14	24.21	48.35	68.29	41.24	Synergist
		2:1	28.96	18.23	47.19	46.34	− 1.80	Additive
		5:1	32.94	10.43	43.37	24.39	− 43.76	Antagonism

## Discussion

Many botanical chemicals with potential insecticide activity have been reported^24^ and only a few studies focused on their sublethal assessments^23^. Rotenone, pyrethrins, matrine, azadirachtin, eucalyptol and extract of *Veratrum nigrum* L., have been registered for aphid control in China. According to our result, the toxicity of rotenone and pyrethrins after treated for 24 and 48 h were near to that of bifenthrin and thiamethoxam, the commonly used chemical insecticides. The acute toxicity of matrine and azadirachtin were much lower than the chemical insecticides. The acute toxicity of eucalyptol, osthole, resveratrol, eugenol, carvacrol, citronellal was the lowest, showing no potential in *R. padi* control by foliar spraying. Ikbal and Pavela summarized essential oils showing high contact toxicity to aphids with LD_50_ lower than 1 µL (µg) mL^−1^ or more than 90% mortality upon application at 2 µL L^−1^. A series essential oils with high toxicity were obtained, including carvacrol to *Lipaphis pseudobrassicae* (Davis)^25^ and *Cerataphis lataniae* (Boisduval)^26^, eucalyptol to *Aphis gossypii* (Glover)^27^, citronellal to *Aulacorthum solani* (Kaltenbach)^28^. The contrastive low toxicity of these botanical insecticides to *R. padi* might be resulted from different aphid species and the different constituents of essential oils. Besides, essential oils containing eugenol as the major constituents showed low contact toxicity to *Phyllaphis fagi* (Linnaeus)*,* either^29^.

Due to the degradation under sunlight and unbalanced spatiotemporal distribution of botanical insecticides when applied by foliar spraying, they might show sublethal effect to *R. padi.* Vania et al. found that exposed to the recommended dosage of botanical insecticide, eucalyptus oil, garlic extract, neem oil, and rotenone, decreased the rate of walking activity in adult workers of honey bees^30^. Zhang et al.^31^ reported that the sublethal concentrations of beta-cypermethrin showed negative impacts on *R. padi* fitness by decreasing pre-adult survival rate and delayed the development of some stages. Here, the low-lethal concentrations (LC_10_ and LC_30_) of rotenone or pyrethrins also showed obvious negative effect on the development and fecundity of the F0 generation, which was consist with most sublethal effects of chemical insecticides to *R. padi*, including dinotefuran^32^, pirimicarb^33^; thiamethoxam^34^. Although botanical insecticides degraded shortly after application and not persisted in the environment or even insect, the negative effect of rotenone and pyrethrins showed transgenerational effect in the F1 generation. This might be related with the embryonic manner of aphids. An aphid has its own embryo since it is born. The embryos of F0 generation were exposed to the low-lethal concentrations of rotenone or pyrethrin with their parental generation. The development duration of nymph in the F1 generation was increased, but the longevity of adult and fecundity was decreased. This might be a fitness cost of insect resulted from the consumption of energy to manage the insecticide suppression and leading to a shortage of energy for productivity.

The sublethal effects of insecticides to the life history and fecundity of individuals might also affect population dynamics^22^. Here, the R_0_, r_m_, λ, GRR decreased significantly after exposed to low-lethal concentrations of rotenone and pyrethrins, while the value of T showed no obvious change. These results suggested that the low-lethal concentrations of rotenone or pyrethrins could suppress the population growth. This phenomenon is similar with the sublethal effect of flonicamid to *Schizaphis graminum* (Rondani)^35^. Exposed to the low-lethal concentrations (LC_5_ and LC_10_) of flonicamid decreased the adult longevity, the fecundity and key demographic parameters (R_0_, r, and λ) in the F1 generation significantly, and also decreased the total duration of feeding on phloem and concurrent salivation in the F0 and F1 generation of *S. graminum* ^35^. However, multiple studies reported a hormesis effect after exposed to the sublethal concentrations of chemical insecticides. Wang et al.^36^ reported that exposed to the sublethal concentrations of sulfoxaflor caused hormesis effects on *A. gossypii* feeding, growth, reproduction behavior, including increasing the fecundity, R_0_ in the F1 generation, and phloem feeding. Exposed to the sublethal concentrations of sulfoxaflor also showed positive effect on the population resurgence of *R. padi*^37^. Similar effect in *R. padi* was reported after exposed to dinotefuran^38^, beta-cypermethrin to *A. glycines*^32^, thiamethoxam to *A. gossypii*^39^, and flupyradifurone to *A. craccivora*^40^. Our results indicated that botanical insecticide rotenone and pyrethrins were not easy to cause pest resurgence and much appropriate for *R. padi* control.

To achieve the reduction and enhanced efficiency of chemical pesticides, and prolonged the control period of botanical insecticide to *R. padi*, synergism combinations containing botanical insecticide and chemical insecticide were screened. Thiamethoxam was suggested to be the best partner to be combined with rotenone or pyrethrins. Mixtures containing rotenone and thiamethoxam or pyrethrins and thiamethoxam both showed synergism effect at most ratios. The co-toxicity factors were also the highest for the mixtures containing thiamethoxam. The LC_50_ value of thiamethoxam to *R. padi* was 16.447 mg/L in this study. According to the susceptibility base line to thiamethoxam (3.6 mg/L) from Gong et al^41^, the *R. padi* population used in this study have developed low resistance to thiamethoxam, which might be accompanied with the fitness cost of energy consumption. Rotenone is an energy inhibitor. The combined application of rotenone could inhibit the energy production and might thus increase its susceptibility. Besides, the target of thiamethoxam, rotenone and pyrethrins are all different and they are suitable to be combined applied. Mixtures of pyrethrins and bifenthrin showed less synergism ratios than the other mixtures, which might be related to their similar target of Na^+^ channel. These results provided suitable combinations for *R. padi* control and could delay the resistance to thiamethoxam.

In conclusion, among the 10 tested botanical chemicals, rotenone and pyrethrins were most valuable to control *R. padi* when applied by foliar spraying. Exposed to the low-lethal concentrations of rotenone or pyrethrins showed negative effect on the development, fecundity, and population growth. Compared with the chemical insecticides with hormesis effect at sublethal concentrations, rotenone and pyrethrins were much more appropriate for *R. padi* control. Mixtures containing rotenone and thiamethoxam, pyrethrins and thiamethoxam at ratios 1:5, 1:2, 1:1 all showed synergist effect.

## Materials and methods

### Insects and Chemicals

The *R. padi* population was provided by the pesticide institute of Henan Institute of Science and Technology. They were kept on wheat seedling without exposure to insecticides and reared in insectary at 22–25 °C, 60–70% relative humidity, 14:10 h of light:dark photoperiod.

The technical grade of rotenone (40%), pyrethrins (50%), matrine (5%), azadirachtin (5%), resveratrol (90%) was provided by Beijing Qingyuanbao Biotechnology Co., LTD (Beijing, China). The high purity of osthole (99%), carvacrol (90%), eugenol (99%) was purchased from aladdin (Shanghai, China). The high purity of eucalyptol (99%) was purchased from J&K (Beijing, China). The technical grade of bifenthrin (97%), thiamethoxam (97%), citronellal (96%) was provided by Henan Jintiandi Co., Ltd (Kaifeng, China).

### Bioassay

The toxicity of insecticides to *R. padi* was determined by leaf-dip method. Briefly, the stock solution (10,000 mg L ^−1^) was prepared using the technical grade insecticides dissolved in acetone, except for matrine, which was dissolved in water. Then, a total of 5–8 diluted concentrations of insecticides were prepared using 0.1% Tween 80. A total of 15 adults of *R. padi* with almost the same size on wheat leaves were dip in the insecticide work solution for 10 s and dried in the air. The treated *R. padi* were transferred to plastic cup laid with 2% agar solution (10 mL) and covered with cling film to prevent escape. Each concentration was conducted in triplicate. The mortality of *R. padi* was checked 24 h and 48 h after treatment. Application of 0.1% Tween 80 was used as control.

### Sublethal effect determination of rotenone and pyrethrins

A total of 30 *R. padi* adults (≤ 24 h old) on fresh wheat leaves was treated with rotenone or pyrethrins at LC_10_ or LC_30_ by dipping in the insecticide solution for 10 s. *R. padi* treated with 0.1% Tween 80 was used as control. The survived adults of *R. padi* (the F0 generation) after treated for 24 h were transferred to the wheat leaves without insecticides treatment and reared in petri dish individually. The fresh wheat leaves were replaced every three days and each treatment included 30 *R. padi* adults*.* The new born *R. padi* (the F1 generation) was checked twice (9:00 a.m. and 21:00 p.m.) every day, and removed timely. The fecundity of the F0 generation was recorded until the *R. padi* adults died naturally. The lifespan of the F0 generation *R. padi* was calculated.

The F1 generation nymph of *R. padi* (≤ 24 h old) laid at the same day under the same low-lethal concentrations was selected and transferred on the fresh wheat leaves without insecticides treatment. Each nymph was reared individually and each treatment included 30 *R. padi* nymphs*.* The fecundity of the F1 generation and their lifespan were recorded as the description above.

### Screening of the synergistic mixture containing botanical insecticide and chemical insecticide

The LC_50_ value of each insecticide in bioassay section was calculated using SPSS 16.0 (SPSS, Chicago, IL, U.S.A.). The synergistic mixtures to *R. padi* were screened using the co-toxicity factor method^42^. The stock solution of insecticides was diluted to LC_50_ and 1/2 LC_50_ using 0.1% Tween 80. Insecticides were mixed well with the volume ratio at 1:5, 1:2, 1:1, 2:1, 5:1, respectively. The adults of *R. padi* were treated with the mixture of insecticides as described in bioassay section. The mortality of adults was recorded 24 h after treatment. The co-toxicity factor was calculated using the following formula:

$${\text{co-toxicity factor}} = \frac{{{\text{observed mortality}} - {\text{expected mortality}}}}{{{\text{expected mortality}}}} \times 100$$

### Statistical analysis

The significant difference of life history and fecundity among the LC_10_, LC_30_ groups and the control group of *R. padi* from F0 and F1 generations were analyzed using one-way analysis of variance (ANOVA) followed by Tukey’s Honestly Significant Difference (HSD) test using SPSS 16.0 (SPSS, Chicago, IL, U.S.A.). *P* < 0.05 was regarded to be statistically significant. Raw data of life history and fecundity of the F1 generation *R. padi* were analyzed according to the age-stage, two-sex life table^43,44^ using TWOSEX-MSChart^45^. The life history parameters (the age-specific survival rate (l_x_), the age-specific fecundity (m_x_), the age-specific maternity (l_x_m_x_), the life expectancy (e_xj_), the age-stage specific reproductive value (V_xj_)), and means and standard error (SE) of population parameters (mean generation time (T), net reproductive rate (R_0_), intrinsic rates of natural increase (r_m_), finite rate of population increase (λ), gross reproduction rate (GRR)) were estimated using 100,000 bootstraps. A paired bootstrap test was performed using TWOSEX-MSChart to compare the differences of population parameters among the low-lethal concentrations (LC_10_ and LC_30_) groups and the control group. The curves of l_x_, m_x_, l_x_m_x_, e_xj_ and V_xj_ were constructed using SigmaPlot 15.0 software. Co-toxicity factor > 20 indicated synergist; − 20 ≤ co-toxicity factor ≤ 20 indicated additive; co-toxicity factor ≤ 20 indicated antagonism.

## Data Availability

Some or all data used during the study are available from the corresponding author by request.
